# Occupation recorded on certificates of death compared with self-report: the Atherosclerosis Risk in Communities (ARIC) Study

**DOI:** 10.1186/1471-2458-7-229

**Published:** 2007-08-31

**Authors:** Aurelian Bidulescu, Kathryn M Rose, Susanne H Wolf, Wayne D Rosamond

**Affiliations:** 1 Cardiovascular Research Institute and Department of Community Health and Preventive Medicine, Morehouse School of Medicine, Atlanta, GA, USA; 2 Department of Epidemiology, School of Public Health, University of North Carolina at Chapel Hill, NC, USA

## Abstract

**Background:**

Death certificates are a potential source of sociodemographic data for decedents in epidemiologic research. However, because this information is provided by the next-of-kin or other proxies, there are concerns about validity. Our objective was to assess the agreement of job titles and occupational categories derived from death certificates with that self-reported in mid and later life.

**Methods:**

Occupation was abstracted from 431 death certificates from North Carolina Atherosclerosis Risk in Communities Study participants who died between 1987 and 2001. Occupations were coded according to 1980 Bureau of Census job titles and then grouped into six 1980 census occupational categories. This information was compared with the self-reported occupation at midlife as reported at the baseline examination (1987–89). We calculated percent agreement using standard methods. Chance-adjusted agreement was assessed by kappa coefficients, with 95% confidence intervals.

**Results:**

Agreement between death certificate and self-reported job titles was poor (32%), while 67% of occupational categories matched the two sources. Kappa coefficients ranged from 0.53 for technical/sales/administrative jobs to 0.68 for homemakers. Agreement was lower, albeit nonsignificant, for women (kappa = 0.54, 95% Confidence Interval, CI = 0.44–0.63) than men (kappa = 0.62, 95% CI = 0.54–0.69) and for African-Americans (kappa = 0.47, 95% CI = 0.34–0.61) than whites (kappa = 0.63, 95% CI = 0.57–0.69) but varied only slightly by educational attainment.

**Conclusion:**

While agreement between self- and death certificate reported job titles was poor, agreement between occupational categories was good. This suggests that while death certificates may not be a suitable source of occupational data where classification into specific job titles is essential, in the absence of other data, it is a reasonable source for constructing measures such as occupational SES that are based on grouped occupational data.

## Background

Data from death certificates are used to monitor age, race and gender variations in mortality in the United States, US [1,2]. While sociodemographic information on death certificates is obtained from next of kin or other proxies, studies have indicated high validity of such information when compared with other official documents [3]. In the late 1980s the National Center for Health Statistics implemented guidelines to standardize data collected on death certificates across the US [4]. As a result information related to employment (job title and industry) and educational attainment is available on certificates of death, which facilitates the monitoring of socioeconomic related trends and rates of mortality across the US In addition, information of employment and education on death certificates is useful in epidemiologic studies when SES is not available from other sources. However, the comparability of such data to that from self-report is not well established.

Studies assessing the agreement of educational attainment from death certificate with that obtained by self- report have reported that death certificates record higher [5,6] and lower [7] levels of education than that obtained by self-report. However, there is high agreement between death certificate-derived educational attainment and that obtained from self-report when data are grouped into ordered categories [5-7]. To our knowledge, the comparability of death certificate-based occupational measures of SES to those obtained by self-report has not been assessed. The purpose of the current study was to compare the agreement of death certificate-based job titles and associated occupational categories with those self-reported in midlife in the Atherosclerosis Risk in Communities (ARIC) Study. We examined agreement overall, and by race, gender, age and educational attainment.

## Methods

Details of the design and procedures of the ARIC Study are presented elsewhere [8]. Briefly, at inception (1987–1989), a biracial cohort of 15,792 middle-aged men and women was sampled from four communities in the United States (Washington County, MD; Forsyth County, NC; north western suburbs of Minneapolis, MN; and Jackson, MS). Institutional review board approval was obtained by each participating field center and the coordinating center. Written informed consent was obtained from each study participant.

Information on current or most recent occupation (if retired) was obtained from the participants at the baseline examination during a standardized interview. Occupations were coded using the corresponding 3 digit code from the 1980 Bureau of Census job titles [9], and the Alphabetical Index of Industries and Occupations [10]. Death certificate-derived occupational data was obtained from 452 ARIC cohort participants from the Forsyth County who died between the baseline examination and 2001. Only Forsyth County participants were included in our investigation because the data originated from a pilot study (an ARIC ancillary investigation) limited to Forsyth County study that included ARIC participants with NC death certificates at their decease. Of these participants, 431 (95%) had occupation recorded on both the death certificates and at the ARIC baseline interview. The decedent's occupation was defined as the usual occupation done during most of his/her working life. Occupations recorded on the death certificates were independently abstracted and coded by two trained coders according to the mentioned 3 digit code from the 1980 Bureau of Census job titles and the Alphabetical Index of Industries and Occupations. When between-coder discrepancies were noted, the coders discussed the discrepancy and attempted to reach agreement. A professional occupational coder adjudicated where agreement could not be reached. Additionally, the professional occupational coder coded a random sample of 45 (10%) death certificates. The percent agreement for the inter-coder variation within the coding process was calculated. Assigned occupational codes were then grouped into 1980 census categories (managerial and professional specialties; technical, sales, and administrative support; service; farming, forestry and fishing; precision production, craft, and repair; and operators, fabricators, and laborers). An additional category was added for homemakers. As an alternative to the census categories, managerial and professional specialties plus technical, sales and administrative support were grouped as "white collar" occupations, whereas all the other categories, except homemakers, were grouped as "blue collar" occupations. In an additional analysis, the time from the ARIC data collection to death was included as a dichotomized stratifying variable, considering the median (2874 days, representing 7.8 years) as the cutpoint.

The occupational data from the death certificates was compared to that self-reported during the ARIC baseline interview. We assessed percent agreement, using standard methods [11], and chance-adjusted agreement by kappa coefficients, with 95% confidence intervals [12]. SAS statistical software version 8.2 was used for the analysis [13].

## Results

The mean age at baseline was 58 years. The average time to death (follow-up time) was 7.7 years. Forty-two percent of decedents were female and 17% were black. Twenty-eight percent had an education that went beyond high school. Between-coder discrepancies in the assigned occupational codes were noted in 13 % (N = 58) of the death certificates. Agreement could not be reached in 23 cases, in which a professional occupational coder adjudicated. Among the random sample of 45 death certificates coded by the professional occupational coder, only one discrepancy with the initial coders was found. For the initial inter-coder variation, the percent agreement for census-based categories, 86.5%, was similar with the comparison death certificate – self-report for the occupational categories.

Agreement between job titles recorded on death certificate to those self-reported was poor (32%). However 67% of census-based occupational categories matched across sources. The kappa coefficient ranged from 0.53 for technical/sales/administrative jobs to 0.68 for homemakers (Table 1). The percent agreement was similar and high across all occupational categories.

**Table 1:** Percentage agreement and chance-adjusted kappa coefficient (95% confidence interval, CI) between self-reported occupational category* and death certificate records by census-based categories

	**Percent Agreement (%)**	**Kappa Coefficient**	**95% C.I.**
**Census-based Categories**			
Managerial/Professional (N^§ ^= 65)	86	0.59	0.54–0.64
Technical/Sales/Administrative (N^§ ^= 53)	85	0.53	0.48–0.58
Service (N^§ ^= 27)	94	0.64	0.60–0.69
Farming/Forestry/Fishing^† ^(N^§ ^= 0)	98	_^†^	_^†^
Precision/Production & Craft/Repair (N^§ ^= 47)	89	0.60	0.56–0.65
Operators/Fabricators/Laborers (N^§ ^= 52)	89	0.62	0.57–0.67
Homemakers (N^§ ^= 43)	92	0.68	0.64–0.73

* Grouped in categories following the 1980 Bureau of Census categories: Managerial and professional specialty; Technical, sales, and administrative support; Service; Farming, forestry and fishing; Precision production, craft, and repair; Operators, fabricators, and laborers; Homemakers.

^§ ^N indicates the number of participants that had the same category on both sources.

^† ^Only five farming/forestry/fishing were self-reported and only three were recorded on death certificates (none one recorded on both sources).

Based on established guidelines [14], the overall chance-adjusted agreement was good, 67% (Table 2). Agreement was nonsignificant lower for women (kappa = 0.54, 95% Confidence Interval, CI = 0.44–0.63) than men (kappa = 0.62, 95% CI = 0.54–0.69) and for African-Americans (kappa = 0.47, 95% CI = 0.34–0.61) than whites (kappa = 0.63, 95% CI = 0.57–0.69). Differences in classification by educational attainment were small. Occupational categories were more likely to agree across sources for decedents who were 56 years of age or older at baseline than for younger participants (Table 2).

**Table 2:** Percentage agreement and chance-adjusted kappa coefficient (95% confidence interval, CI) between self-reported occupational category* and death certificate records by selected characteristics

	**Percent Agreement (%)**	**Kappa Coefficient**	**95% C.I.**
**All **(N = 431)	67	0.60	0.55–0.65
			
**Sex**			
Men (N = 247)	70	0.62	0.54–0.69
Women (N = 184)	65	0.54	0.44–0.63
			
**Ethnicity**			
Whites (N = 348)	70	0.63	0.57–0.69
African-Americans (N = 83)	57	0.47	0.34–0.61
			
**Education**^§^			
Low and medium (N = 305)	66	0.59	0.52–0.65
High (N = 126)	69	0.54	0.43–0.66
			
**Age**			
45–50 (N = 54)	60	0.51	0.35–0.67
51–55 (N = 79)	62	0.53	0.46–0.60
56–60 (N = 144)	72	0.64	0.59–0.69
61–65 (N = 154)	67	0.60	0.52–0.68
			
**"Collar-type" Categories†**			
"White Collar" Occupations (N^‡ ^= 153)	87	0.74	0.70–0.78
"Blue Collar" Occupations (N^‡ ^= 161)	86	0.72	0.68–0.76
			
**Time to Death**			
Lower than 7.8 yrs (N = 218)	69	0.63	0.56–0.70
Greater or equal than 7.8 yrs (N = 213)	64	0.56	0.48–0.64

* Grouped in categories following the 1980 Bureau of Census categories: Managerial and professional specialty; Technical, sales, and administrative support; Service; Farming, forestry and fishing; Precision production, craft, and repair; Operators, fabricators, and laborers; Homemakers.

^§ ^Defined as Low and medium for 12 years or less, and High for more than 12 years.

^† ^"Collar-type" categories were defined as the following groups: managerial and professional specialties plus technical, sales and administrative support as "white collar"; all other categories, except homemakers, as "blue collar".

^‡ ^N indicates the number of participants that had the same category on both sources.

As expected, when occupations were grouped into "white collar" – "blue collar" occupations, the chance-adjusted kappa coefficient between the two sources was higher that with census-based categories (Table 2). In the analysis that incorporated the time from the ARIC baseline examination to death as a stratifying variable, those who died earlier had a higher kappa coefficient than those who survived longer (Table 2).

The job titles that were found most frequently in the death certificate-derived occupational data were as follows. The study participants were administrative assistant (in 3 cases), agent (3), clerk (10), contractor (3), electrician (4), engineer (5), inspector (9), machine operator (13), machinist (4), maintenance (4), manager (7), mechanic (13), minister (4), owner/operator (12), plumber (3), salesman (3), secretary (5), supervisor (10), teacher (13), truck driver (16) and worker in the tobacco products manufacturing (4).

## Discussion

We found that the agreement of occupational titles recorded on death certificates to those self-reported between the ages of 45 and 64 years was poor. This is consistent with other studies based mostly on occupational cohorts that reported poor to fair agreement between death certificate-derived job titles to those obtained from occupational records and other proxy reports [15-20]. However, when death-certificate derived job titles were grouped into standard census occupational categories, agreement with categories based on self-reported occupation at midlife was good. The kappa coefficient was similarly high across occupational categories.

Studies assessing concordance of occupations recorded on death certificates to those reported in employment records tend to report low to fair agreement. However, few studies have assessed the concordance of occupational categories typically used to measure SES. The different modalities used to capture occupation (current/last for midlife interview versus usual/most of his or her life, for death certificate) does not seem to produce a large difference, as illustrated by similar agreement at different age groups. Nevertheless, age differences exist, suggesting that a cohort effect is possible. This could be explained by the observation that people from earlier birth cohorts had less occupational mobility, similar perhaps nowadays with that of women and African-Americans. Alternatively, it may be explained by recall error on part of the proxy. Proxies may elevate the occupation prestige of decedents. The question remains if midlife occupation is representative of the occupation during one's work life. This assumption is very important when assessing accuracy of the information from death certificates. To our best knowledge there are no related results from previous studies.

Our study has several limitations. We included data from only one geographical area (state). However, since death certificates across the US are standardized to include usual occupation across life, substantial variation across states should not be expected. Also, our decedents are from limited birth cohorts (1920s–1940s), which may limit inferences to different time periods. There could be secular differences in that population expectancy for a job or special issues as the women in those birth cohorts were often homemakers. The lack of agreement could also in part reflect differences in the type of occupations held at midlife versus what the decedent was doing most of his/her work life. Another important limitation of the study is that information from the two sources was not assessed at the same time, and the inconsistency is not only related to the reporter (self versus proxy) but also to the length of time between the study baseline and death.

Among the advantages of our study are the inclusions of African-American and female participants, and the utilization of a standardized approach to code job titles. Another advantage of our study is that 95% of the ARIC participants, which died between 1987 and 2001, had information on occupation recorded on both the death certificate and the ARIC study questionnaire. This represents a unique aspect of our investigation, since high percentage of missing socioeconomic status information on death certificate data can limit usage of SES recorded on death certificate.

Our study invites similar investigations in different populations within and outside the United States in order to confirm the potential significance and generalizability of our results.

## Conclusion

Our study is consistent with other studies suggesting that death certificates may not be an appropriate source of occupational data when information on exposure to specific jobs is essential. However, our findings suggest that they may be a reasonable source for measures such as occupational socioeconomic status that are based on grouped occupational data.

## Competing interests

The author(s) declare that they have no competing interests.

## Authors' contributions

AB and KMR conceived of and designed the study. AB performed the statistical analyses. AB, KMR and WDR interpreted the results. AB and KMR drafted the manuscript. All authors revised the manuscript for intellectual content, and read and approved the final manuscript.

## Pre-publication history

The pre-publication history for this paper can be accessed here:


